# Chronic exposure to inhaled vaporized cannabis high in Δ9-THC alters brain structure in adult female mice

**DOI:** 10.3389/fnins.2023.1139309

**Published:** 2023-03-06

**Authors:** Autumn Taylor, Amanda Nweke, Veniesha Vincent, Marvellous Oke, Praveen Kulkarni, Craig F. Ferris

**Affiliations:** ^1^Department of Biology, Morgan State University, Baltimore, MD, United States; ^2^Center for Translational NeuroImaging, Northeastern University, Boston, MA, United States; ^3^Department of Psychology and Pharmaceutical Sciences, Northeastern University, Boston, MA, United States

**Keywords:** diffusion weighted imaging, voxel-based morphometry, dopaminergic system, prefrontal cortex (PFC), limbic cortex

## Abstract

**Introduction:**

The medical and recreational use of cannabis has increased in the United States. Its chronic use can have detrimental effects on the neurobiology of the brain—effects that are age-dependent. This was an exploratory study looking at the effects of chronically inhaled vaporized cannabis on brain structure in adult female mice.

**Methods:**

Adult mice were exposed daily to vaporized cannabis (10.3% THC and 0.05% CBD) or placebo for 21 days. Following cessation of treatment mice were examined for changes in brain structure using voxel-based morphometry and diffusion weighted imaging MRI. Data from each imaging modality were registered to a 3D mouse MRI atlas with 139 brain areas.

**Results:**

Mice showed volumetric changes in the forebrain particularly the prefrontal cortex, accumbens, ventral pallidum, and limbic cortex. Many of these same brain areas showed changes in water diffusivity suggesting alterations in gray matter microarchitecture.

**Discussion:**

These data are consistent with much of the clinical findings on cannabis use disorder. The sensitivity of the dopaminergic system to the daily exposure of vaporized cannabis raises concerns for abuse liability in drug naïve adult females that initiate chronic cannabis use.

## Introduction

With the gradual legalization and social acceptance of cannabis in the United States, its risk for abuse has increased significantly (Hasin et al., 2015; Carliner et al., 2017). A recent study by Carlini and Schauer (2022) reported the age-related prevalence of cannabis use among US adults. Adolescent to young adults, 18–25 years old, have the highest prevalence (2.0%) of cannabis use-only followed by 26–49 years old (0.7%), and 0.6% for over 50 years of age (Carlini and Schauer, 2022). Whereas 9.4% of the general population use cannabis with other drugs like nicotine and alcohol. In the United States there are more male users of cannabis than female (Khan et al., 2013; Ketcherside et al., 2016), although women seem to be more prone to cannabis use disorder (CUD) (Hernandez-Avila et al., 2004; Cooper and Craft, 2018). Seventeen is the average age for a first-time user of cannabis (Korf et al., 2007).

The major psychoactive molecule in cannabis plant is Δ9-tetrahydrocannabinol (Δ9-THC) (Mechoulam et al., 2014). The primary target for Δ9-THC is the CB1 receptor that is found in high density in the caudate/putamen, hippocampus, prefrontal cortex and cerebellum (Herkenham et al., 1991). There are differences in brain morphology and cognitive function between adolescents and adults that smoke cannabis high in Δ9-THC (Batalla et al., 2013). This is not surprising since the adolescent brain is still undergoing maturational organization, particularly in the area of the prefrontal cortex (Rubino et al., 2015). Adolescent rodents exposed to repeated Δ9-THC injections present with long-term deficits in cognition (Silva et al., 2016; Murphy et al., 2017). Chronic Δ9-THC injections have also been shown to cause longer-lasting memory deficits in adolescent mice but not in adults (Kasten et al., 2017). Adolescent male and female rats exposed to cannabis smoke or Δ9-THC present with subtle changes in cognitive and emotional behavior (Bruijnzeel et al., 2019). Coleman et al. (2022) exposed adolescent male and female mice to inhaled vaporized cannabis high in Δ9-THC for 28 days and reported sex difference in morphology and brain function. Females showed changes in gray matter microarchitecture in the prefrontal cortex and accumbens while males showed altered functional connectivity in hippocampal circuitry.

While there have been many studies on adults ranging from 18 to 40 years of age, the participants have a long history of cannabis use, so it is not possible to discern the effects of chronic cannabis exposure on the drug naïve, adult brain. In a recent study Sadaka et al. (2023) exposed old female mice, 19–20 months of age, to vaporized cannabis high in THC (10.3%) for 28 days and reported structural changes in the dopaminergic (DA) system. This finding is evidence that the drug-naïve, aged brain can make neuroadaptive changes to repeated exposure to inhaled cannabis and raises the possibility of abuse liability in the elderly population. This notion, of cannabis-induced neuroadaptation in the mature, drug-naïve brain was evaluated in this study using adult female mice. We hypothesized that these mice would also present anatomical changes in neural circuitry associated with DA neurotransmission as evaluated with voxel-based morphometry and diffusion weighted imaging MRI.

## Materials and methods

### Animal usage

Female c57bl/j6 mice (*n* = 24) were obtained from Charles River Laboratories, (Wilmington, MA, USA). The mice were ca 130 days of age at the start of the experiment to ensure they were mature adult animals (Brust et al., 2015). All mice were housed in groups of four, maintained on a 12:12 h light-dark cycle with lights off at 19:00 h, and allowed access to food and water *ad libitum*. All mice were acquired and cared for in accordance with the guidelines published in the Guide for the Care and Use of Laboratory Animals (National Institutes of Health Publications No. 85–23, Revised 1985) and adhered to the National Institutes of Health and the American Association for Laboratory Animal Science guidelines. The protocols used in this study comply with the regulations of the Institutional Animal Care and Use Committee at Northeastern University and adhere to the ARRIVE guidelines for reporting *in vivo* experiments in animal research (Kilkenny et al., 2010).

### Cannabis exposure

Mice (*n* = 12) were exposed to cannabis high in THC (10.3% THC and 0.05% CBD), or placebo cannabis (*n* = 12) with less than 0.01%THC and 0.01% CBD. Cannabis was acquired from the National Institute on Drug Abuse (NIH/NIDA, Bethesda, MD, USA) through the Research Triangle Institute (Research Triangle Park, NC, USA). Groups of mice were placed in a 38-L exposure chamber (60 cm × 45 cm × 20 cm), that included a vapor inflow tube and several small air outflow holes. Subjects were acclimated to the exposure environment for two days prior to exposure to reduce any stress of the novel environment. A Volcano Vaporizer (Storz and Bickel, Tuttlingen, Germany) was used to heat cannabis plant material below the point of complete combustion to vaporize the active ingredient (Δ9-THC), minimizing the generation of harmful free radicals such as polycyclic aromatic hydrocarbons associated with the combustion of organic plant material. The vaporizer was preheated at approximately 210°C and loaded with 0.450 g of minced cannabis. Tubing was attached from the vaporizer to the exposure chamber and the heating fan was run for a total of 60 s, filling the exposure chamber with vaporized cannabis aerosols. After 30 min of passive exposure, mice were removed from the exposure chamber and returned to their cages. This exposure protocol occurred daily for 21 consecutive days. The mass of minced cannabis was based on a previously published study showing that this approach yielded similar serum Δ9-THC concentrations (130–150 ng/ml) to those reported in human users (Farra et al., 2020). Mice were imaged within 48 h after the last exposure.

### Neuroimaging

Imaging was done using a Bruker Biospec 7.0T/20-cm USR horizontal magnet (Bruker, Billerica, MA, USA) and a 20-G/cm magnetic field gradient insert (ID = 12 cm) capable of a 120-μs rise time. Radio frequency signals were sent and received with a quadrature volume coil built into the animal restrainer (Ekam Imaging Inc., Boston, MA, USA). The design of the restraining system included a padded head support obviating the need for ear bars helping to reduce animal discomfort while minimizing motion artifact (Ferris et al., 2014; Ferris, 2022). All mice were imaged while under light 1% isoflurane anesthesia for a maximum of one hour. The respiration rate was ca 50–55 breaths/min. At the beginning of each imaging session, a high-resolution anatomical data set was collected for volumetric analysis using a RARE (Rapid Acquisition with Relaxation Enhancement) pulse sequence with the following parameters: 20 slices of 0.7 mm thickness; field of view (FOV) 3 cm; 128 × 128; a repetition time (TR) of 3,000 ms; an effective echo time (TE) of 32 ms, and number of averages (NEX) of 5 acquisition, for a total time 3 min 20 s.

### Voxel based morphometry

Voxel based morphometry is a technique to look for structural changes in brain anatomy scans. It is data driven technique where whole brain scans are registered to a template, i.e., atlas, and based on atlas information segmented into various brain volumes. This technique was used to see if there were changes in brain volumes in different brain areas with chronic vaporized cannabis exposure versus placebo. A 3D Mouse MRI Brain Atlas©with 139 segmented and annotated brain regions (Ekam Solutions; Boston, MA, USA) was used to calculate brain volumes, and register the standard structural mouse template image onto the high resolution T2-weighted images for each individual subject using a non-linear registration method implemented by Unix based software package Deformable Registration via Attribute Matching and Mutual-Saliency Weighting (DRAMMS).^1^ The atlas (image size 256 x 256 x 63) was then warped from the standard space into the subject image space (image size 128 × 128 × 20) using the deformation obtained from the previous step and the nearest-neighbor interpolation method. In the volumetric analysis, each brain region was therefore segmented, and the volume values extracted for all 139 ROIs, calculated by multiplying unit volume of voxel in mm^3^ by the number of voxels using an in-house MATLAB script (available upon request). To account for different brain sizes all the ROI volumes were normalized by dividing each ROI volume by total brain volume of that subject. Differences in brain volumes (mm^3^) between 139 areas were compared across each of the two experimental conditions using a non-parametric Kruskal-Wallis test (alpha set at 5%). All volumetric data are provided in Supplementary data file 1.

### Diffusion weighted imaging—Quantitative anisotropy

Diffusion weighted imaging (DWI) can be used to assess changes in gray matter microarchitecture that may result from alterations in extracellular and intracellular water, numbers of glia, neurons, dendrites and axons, capillary density, connective tissue and perineuronal nets. DWI provides a numerical measure of how water moves under these different conditions. DWI was acquired with a spin-echo echo-planar-imaging (EPI) pulse sequence having the following parameters: TR/TE = 500/20 ms, eight EPI segments, and 10 non-collinear gradient directions with a single b-value shell at 1,000 s/mm^2^ and one image with a B-value of 0 s/mm^2^ (referred to as B0). Geometrical parameters were: 48 coronal slices, each 0.313 mm thick (brain volume) and with in-plane resolution of 0.313 × 0.313 mm^2^ (matrix size 96 × 96; FOV 30 mm^2^). Image reconstruction included DWI analysis of the DW-3D-EPI images to produce the maps of apparent diffusion coefficient (ADC). DWI analysis was implemented with MATLAB and MedINRIA^2^ software. Because sporadic excessive breathing during DWI acquisition can lead to significant image motion artifacts that are apparent only in the slices sampled when motion occurred, each image (for each slice and each gradient direction) was screened, prior to DWI analysis, for motion artifacts; if found, acquisition points with motion artifacts were eliminated from the analysis. In these studies two mice from the placebo group and one mouse from the THC cannabis group were removed.

For statistical comparisons between mice, each brain volume was registered to the mouse atlas allowing voxel- and region-based statistics. All image transformations and statistical analyses were conducted using the in-house EVA software. For each mouse, the B0 image was co-registered with the brain atlas (using affine transformation). The co-registration parameters were then applied to the DWI indexed maps for the different indices of anisotropy. Average value and its standard deviation for each region of the brain was computed from map files. Statistical differences in measures of DWI between experimental groups were determined using Student t-test (alpha set at 5%). The formula below was used to account for false discoveries from multiple comparisons for both VBM and DWI.


$$P\,\left(i\right)\leq \frac{i}{V}\,\frac{q}{c\,\left(V\right)}$$

*P*(*i*) is the i-value based on the t-test analysis. Each of 139 ROIs (i) within the brain containing (V) ROIs was ranked in order of its probability value (see Tables 1, 2). The false-positive filter value q was set to 0.2 and the predetermined c(V) set at unity. All DWI data is provided in Supplementary data file 2.

**TABLE 1:** Voxel based morphometry.

Voxel based morphometry (mm^3^)								
	**Placebo**			**Cannabis**				
**Brain area**	**Med**	**Min**	**Max**	**Med**	**Min**	**Max**	***p*-Val**	**Ω^2^**
Primary somatosensory ctx	21.70	19.12	27.09	>18.08	16.81	19.73	9E-05	0.646
Visual 1 ctx	15.96	14.11	17.95	>12.99	10.91	14.77	0.0003	0.526
Fimbria hippocampus	3.02	2.10	3.68	>1.95	1.56	2.96	0.0008	0.454
Rostral piriform ctx	9.13	6.67	10.81	>6.99	5.99	7.77	0.0015	0.403
Retrosplenial rostral ctx	5.23	4.78	6.28	>4.23	3.38	5.43	0.0018	0.387
Corpus callosum	5.38	4.24	6.52	>4.67	4.29	5.08	0.0024	0.362
Frontal association ctx	3.61	2.71	7.90	>2.74	2.24	3.70	0.0027	0.355
Secondary motor ctx	7.15	5.37	9.84	>5.76	5.11	6.59	0.0032	0.339
Secondary somatosensory ctx	5.39	4.71	7.35	>4.89	4.06	5.51	0.0039	0.324
Primary motor ctx	6.57	4.83	8.49	>5.18	4.47	5.76	0.0047	0.309
Posterior thalamic area	1.51	1.23	1.62	<1.71	1.38	2.24	0.0047	0.309
Endopiriform area	2.08	1.56	2.26	>1.73	1.29	2.06	0.0051	0.302
Medial preoptic area	1.36	0.73	1.77	>1.05	0.61	1.62	0.0056	0.295
Temporal ctx	1.79	1.01	2.39	>1.31	0.82	1.81	0.0066	0.281
Periaqueductal gray	6.15	4.07	6.85	>4.99	3.42	5.73	0.0066	0.281
Orbital ctx	5.91	4.15	13.19	>4.33	3.18	5.79	0.0072	0.274
Vestibular area	1.83	0.50	2.49	<2.20	1.87	3.99	0.0086	0.260
Anterior thalamic area	1.48	0.61	1.74	>1.10	0.72	1.37	0.0093	0.254
Paraventricular hypothalamus	0.29	0.14	0.44	>0.20	0.14	0.30	0.0099	0.249
Cerebellar nuclear area	1.15	0.47	1.66	<1.66	1.16	2.39	0.011	0.240
Caudate putamen	18.46	16.24	23.80	>16.97	14.95	18.42	0.0111	0.240
Insular caudal ctx	2.52	2.28	3.52	>2.29	1.79	2.95	0.014	0.222
10th cerebellar lobule	0.72	0.00	1.19	<1.13	0.58	2.16	0.0152	0.215
Insular rostral ctx	5.19	3.77	6.53	>4.29	3.60	5.11	0.0193	0.196
Ventral pallidum	2.27	1.54	3.82	>1.97	1.29	2.45	0.0208	0.191
Auditory ctx	3.94	2.78	4.57	>3.18	2.92	3.96	0.0209	0.191
Caudal piriform ctx	5.84	4.51	6.63	>5.54	4.36	6.22	0.0243	0.179
Parvicellular reticular area	1.17	0.64	1.97	<1.67	0.86	2.42	0.0243	0.179
Reuniens thalamic area	0.60	0.42	0.80	>0.49	0.32	0.65	0.0259	0.174
External capsule	4.05	3.35	4.58	>3.84	3.18	4.19	0.0281	0.168
Claustrum	0.72	0.47	1.63	>0.60	0.36	0.75	0.0345	0.152
Anterior olfactory area	7.13	5.07	13.59	>6.15	4.96	7.02	0.0376	0.146

**TABLE 2:** Apparent diffusion coefficient.

Apparent diffusion coefficient								
	**Placebo**			**Cannabis**				
**Brain area**	**Med**	**Min**	**Max**	**Med**	**Min**	**Max**	***p*-Val**	**Ω^2^**
Accumbens shell	1.09	0.95	1.12	<1.27	1.12	1.41	0.0001	0.702
Endopiriform area	1.15	1.06	1.23	<1.32	1.20	1.47	0.0002	0.671
Accumbens core	1.09	0.94	1.18	<1.27	1.11	1.37	0.0003	0.630
Medial septum	1.18	1.01	1.33	<1.38	1.21	1.58	0.0005	0.576
Secondary motor ctx	2.23	1.68	2.76	>1.80	1.43	2.04	0.0006	0.550
Ventral pallidum	1.18	1.07	1.26	<1.31	1.15	1.46	0.0007	0.540
Parafascicular thalamus	1.24	1.11	1.27	<1.29	1.11	1.53	0.0016	0.457
Globus pallidus	1.19	1.07	1.24	<1.26	1.14	1.36	0.0017	0.455
Primary motor ctx	1.93	1.60	2.64	>1.65	1.35	1.99	0.0024	0.419
Retrosplenial rostral ctx	2.35	2.11	2.71	>1.95	1.59	2.40	0.0024	0.419
Parietal ctx	2.35	1.99	2.77	>1.98	1.57	2.37	0.0025	0.418
Frontal association ctx	1.86	1.32	2.58	>1.61	1.34	1.83	0.0038	0.376
Anterior commissure	1.18	1.02	1.32	<1.31	1.23	1.42	0.0048	0.355
Rostral piriform ctx	1.28	1.13	1.56	<1.42	1.31	2.08	0.0054	0.344
Claustrum	1.19	1.06	1.34	<1.32	1.17	1.42	0.0067	0.324
Ventral thalamus	1.26	1.17	1.29	<1.31	1.18	1.46	0.0089	0.297
Primary somatosensory ctx	1.80	1.46	2.02	>1.57	1.30	1.83	0.0111	0.277
Tenia tecta ctx	1.39	1.16	1.65	<1.63	1.46	2.15	0.0137	0.258
Lateral preoptic area	1.23	1.06	1.46	<1.39	1.18	1.63	0.015	0.250
Anterior cingulate ctx	1.87	1.57	2.42	>1.62	1.31	2.01	0.0201	0.223
Gigantocelllaris reticular area	1.68	1.40	2.31	>1.35	1.14	1.92	0.022	0.215
Diagonal band of Broca	1.24	1.08	1.58	<1.43	1.20	2.13	0.024	0.207
Extended amydala	1.22	1.10	1.36	<1.30	1.16	1.41	0.0241	0.207
Prelimbic ctx	1.77	1.38	2.66	>1.60	1.24	1.76	0.0242	0.207
Bed nucleus stria terminalis	1.30	1.07	1.41	<1.39	1.23	1.52	0.0262	0.200
Intermediate reticular area	1.58	1.35	1.91	>1.37	1.13	1.75	0.0288	0.191
Insular rostral ctx	1.22	1.08	1.53	<1.34	1.16	1.76	0.0345	0.175
Medial mammillary area	1.63	1.40	1.90	>1.49	1.17	1.84	0.0377	0.168
Corpus callosum	1.82	1.71	2.01	>1.68	1.49	2.29	0.0405	0.162
Reticulotegmental nucleus	1.61	1.41	1.74	>1.39	1.18	1.79	0.0409	0.161
10th cerebellar lobule	1.33	1.08	1.90	<1.67	1.19	2.00	0.0409	0.161
Ventral medullary reticulum	1.80	1.34	3.10	>1.44	0.00	1.97	0.0409	0.161
Caudate putamen	1.27	1.15	1.33	<1.34	1.22	1.43	0.0443	0.154
Infralimbic ctx	1.25	1.07	1.63	<1.41	1.17	1.54	0.0483	0.146

## Results

### Voxel based morphometry

**FIGURE 1:**
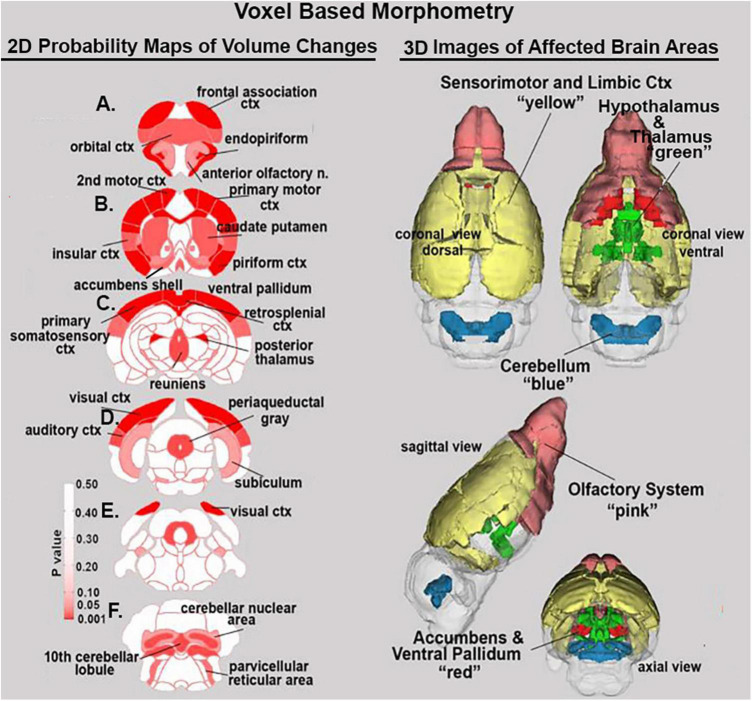
Voxel based morphometry. The location of many of the brain areas listed in Table 1 are shown in the 2D heat maps and are summarized in the 3D color-coded reconstructions to the right.

Shown Table 1 is a list of 32/139 brain areas that were significantly different between placebo and cannabis after 21 days of exposure to each vaporized sample. The brain areas are ranked in order of their significance (α < 0.05) with a false discovery rate of *p* = 0.046. The median (Med) volumes (mm^3^) are highlighted in gray for each brain area along with p-values. Effect sizes ranged from 0.646 and 0.526 for somatosensory and visual cortices, down to 0.152 and 0.146 for claustrum and anterior olfactory area (see Supplementary data file 2). A majority of brain areas were smaller in volume following exposure to cannabis high in Δ9-THC with the exception of brain areas localized to the brainstem and cerebellum (e.g., parvicellular reticular area, cerebellar nuclei, 10th cerebellar lobule, vestibular area). Figure 1 shows probability heat maps marking the location of brain areas significantly different in volume between placebo and cannabis. These data were taken from Table 1 and are aligned top (rostral to bottom (caudal) as 2D axial sections using the 3D mouse MRI atlas. Note the forebrain (sections A and B) comprising the frontal association ctx, orbital ctx, and 2nd motor cortex and brain areas with dopaminergic inputs (section B), e.g., caudate/putamen and ventral pallidum, are reduced in volume. Numerous cortical areas were also reduced in volume (sections A–E) e.g., primary motor, piriform, insular, somatosensory, visual, and auditory cortices. The most caudal brain areas (section F) increased in volume as noted above. The data from Table 1 and the 2D maps are summarized in the 3D color-coded volumes displayed in a mouse “glass” brain from different viewpoints.

### Diffusion weight imaging

**FIGURE 2:**
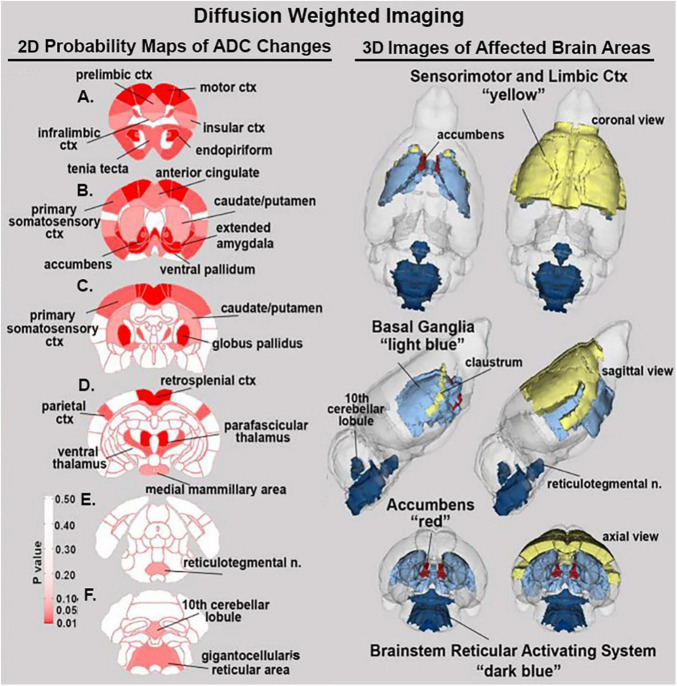
Diffusion weighted imaging. The location of many of the brain areas listed in Table 2 are shown in the 2D heat maps and are summarized in the 3D color-coded reconstructions to the right.

Shown in Table 2 is a list of 34/139 brain areas that were significantly different in ADC values between placebo and cannabis. The brain areas are ranked in order of their significance (α < 0.05) with a false discovery rate of *p* = 0.051. Effect sizes ranged from 0.702 and 0.630 for accumbens shell and core, down to 0.154 and 0.146 for caudate/putamen and infralimbic ctx (see Supplementary data file 2). The median (Med) ADC values are highlighted in gray for each brain area along with *p*-values. There was no consistent change in ADC values between placebo and cannabis. Brain areas associated with the dopaminergic system, e.g., accumbens, ventral pallidum, globus pallidus, caudate putamen, present with higher ADC values while areas associated with the cerebrum, e.g., 2nd motor, primary motor, parietal, somatosensory, retrosplenial, prelimbic, and anterior cingulate cortices, show lower ADC values with cannabis. Figure 2 shows probability heat maps marking the location of brain areas significantly different in ADC values between placebo and cannabis reported in Table 2. The forebrain (sections A and B) shows cannabis-induced changes in the limbic cortex, e.g., prelimbic, infralimbic, insular, and anterior cingulate cortices, together with brain areas comprising the DA system (sections B & C). The caudal most brain areas (sections E and F) would appear less sensitive to the effects of chronic effects of inhaled cannabis high in Δ9-THC. The data from Table 2 and the 2D maps are summarized in the 3D color-coded volumes in Figure 2.

## Discussion

It is well established that chronic cannabis use has deleterious effects on the younger population (Sagar and Gruber, 2018). Cannabis alters brain morphology and function leading to poorer working memory and greater impulsivity (Crane et al., 2013). Clinical and translational research has associated chronic cannabis exposure with alterations in cerebellar gray matter, resting-state functional connectivity, and hippocampal volumes contributing to problems in cognition (Yucel et al., 2008;Ashtari et al., 2011; Batalla et al., 2013; Dahlgren et al., 2016; Blithikioti et al., 2019). Here we show that fully mature, adult female mice show changes in brain morphology in response to chronic inhaled cannabis. These changes are discussed with respect to age- and sex-dependent sensitivity to cannabis in rodents and the parallels to the human condition.

### Voxel based morphometry

In a recent study, we exposed periadolescent male and female mice to inhaled vaporized cannabis for 28 days starting from postnatal day 23 to postnatal day 51 using the same cannabis sample (10.3% Δ9-THC) and procedure used here on adult female mice (Coleman et al., 2022). Imaging data were acquired within 48 hrs after cessation of cannabis exposure as described in this study. We found no significant volumetric changes for either males or female mice in any of the 139 brain areas studied using the mouse 3D MRI atlas. In contrast, adult female mice in this study show numerous volumetric changes in the cerebrum and forebrain in response to chronic cannabis exposure. This would suggest the effects of chronic cannabis use are influenced by brain development, maturation, and age. Whether the changes we found in adult females would persist following an extended period of abstinence is unknown. Our laboratory extended these studies on chronic cannabis exposure and age-related changes in brain neurobiology to include very old female mice (Sadaka et al., 2023). Again using the same cannabis sample, vaporization procedure and exposure duration, we found no volumetric changes in the brain with the exception of the DA system as discuss below.

Much of the human volumetric imaging data on adult volunteers with a history of cannabis abuse are equivocal (Nader and Sanchez, 2018). The data is most consistent around the hippocampus were males with a history of chronic cannabis use present with reduced hippocampal volume and gray matter density (Matochik et al., 2005; Yucel et al., 2008; Demirakca et al., 2011; Cousijn et al., 2012; Wang et al., 2021a). There are no reports of sex differences in the hippocampus or any other brain areas in adult cannabis users (Ketcherside et al., 2016; Nader and Sanchez, 2018) with the possible exception of the cerebellum. The present study on adult female mice found no volumetric differences in any of the hippocampal subregions, e.g., CA3, CA1, or dentate gyrus. Female mice did show an increase in cerebellum volume, albeit limited to only the deep cerebellar nuclei and the 10th cerebellar lobule. This is in contrast to the reported decrease in the volume of the cerebellar cortex in women with a history of cannabis use (McPherson et al., 2021). However, clinical studies composed primarily of men report an increase in cerebellar volume and gray matter density with chronic cannabis use (Cousijn et al., 2012; Battistella et al., 2014; Moreno-Rius, 2019; Wang et al., 2021b). In contrast, Battistella et al. (2014) reported long-term cannabis use in healthy men 18–30 years of age was also associated with a reduction in gray matter volume in many areas of the cerebral cortex as shown here with adult female mice. These authors suggested the change in volume was mediated by the high density of CB1 receptors in the human cortex (Herkenham et al., 1990) which is also true in mice (Miederer et al., 2020).

### Diffusion weighted imaging

The aforementioned study on male and female mice exposed to vaporized cannabis throughout periadolescence found sex-dependent changes in measure of water diffusivity, a surrogate marker of gray matter microarchitecture (Coleman et al., 2022). Females showed lower ADC values in the forebrain, prefrontal cortex, and olfactory system while at the same time presenting with higher ADC values in the hindbrain cerebellum and brainstem reticular activating system. The present study on adult female mice also found ADC changes in numerous forebrain areas and the olfactory system but only a few changes in the hindbrain. However, in very old female mice there were no significant changes in ADC values with chronic cannabis exposure (Sadaka et al., 2023). Higher or lower values of ADC usually reflect an increase or decrease, respectively, in extracellular water and have been used to follow subtle changes in gray matter microarchitecture with head injury (Kulkarni et al., 2015). The use of DWI to characterize the effects of cannabis use in humans has primarily been limited to analysis of white matter pathways providing evidence of impaired connectivity in the developing brain (Zalesky et al., 2012). In this study, the anterior commissure, and the corpus collosum showed significantly altered ADC values in adult female mice.

As shown in Figure 2, adult females exposed to cannabis show ADC changes in the limbic cortex, e.g., anterior and retrosplenial cortices, prelimbic and infralimbic cortices and insular cortex. The alterations in gray matter microarchitecture may reflect a change in the neural circuits regulating emotional behavior and experience. Chronic exposure to vaporized cannabis also affected brain areas associated with the ascending reticular activating system e.g., gigantocellularis reticular area, reticulotegmental area, ventral medullary reticulum, and intermediate reticular area. These areas are associated with arousal and sleep/waking.

### Drug liability?

All drugs of abuse influence the activity of the DA system to affect neuroadaptive changes involved in drug reinforcement (Koob and Volkow, 2016). The chronic use of cannabis increases the risk of substance abuse and dependence (Ramesh et al., 2011; Volkow et al., 2014). Repeated exposure to Δ9-THC in mice and rats also leads to physical dependence ((Wilson et al., 2006; Manwell et al., 2014; Bruijnzeel et al., 2016). Freels et al. (2020) reported that vaporized cannabis extracts have reinforcing properties and support conditioned drug-seeking behavior in rats. Both VBM and DWI in this study on adult female mice revealed changes in the accumbens, ventral pallidum and caudate putamen, key brain areas high in afferent connections from the midbrain DA system. The sensitivity of these areas to Δ9-THC is consistent with the preclinical literature (Kolb et al., 2006; Madularu et al., 2017). Interestingly, the Coleman study on periadolescent mice exposed to vaporized cannabis found no change in the DA system with VBM and a modest change in ADC values, and only then to the accumbens core (Coleman et al., 2022). The Sadaka et al. (2023) study on old female mice found a significant reduction in volume in the DA system but no change in ADC values in response to chronic cannabis exposure.

### Limitations

As an exploratory study, there are many limitations and unanswered questions. First, the study only involved females. Would males have shown the same changes in measures of VBM and DWI? The recent study by Sadaka et al. (2023) on old mice was composed of 19 females and 4 males. There were no obvious differences between the two sexes, so while the sample was very small there is no reason to assume a sex difference in Δ9-THC induced neuroplasticity in adult animals exposed to vaporized cannabis. While the DA system was significantly affected by chronic cannabis exposure in this study on mature females there were no behavioral studies to evaluate dependence. Indeed, the study could have benefited from a battery of behavioral tests for cognition, motor control and nociception. Blood levels of THC were not measured but assumed to be comparable to those reported by Farra et al. (2020), in male mice using the same inhalation and vaporization methods and mass of 10.3% THC cannabis but not taking into consideration sex differences in THC pharmacokinetics. Postmortem histochemistry could have helped to understand the mechanism(s) behind the changes in ADC values. Lastly, the exposure of vaporized cannabis over a period of 21 days in an adult mouse is comparable to 2.3 years in humans (Dutta and Sengupta, 2016). Hence one must consider the final drug-induced effects as an interaction between an aging brain and Δ9-THC, together with the other bioactive phytocannabinoids and terpenoids in the cannabis sample.

### Summary

This was an exploratory study looking at the neuroradiological effects of chronic cannabis exposure on adult female mice. There were volumetric changes in discrete brain areas that included the prefrontal cortex, olfactory and DA systems. Many of these same brain areas showed changes in ADC values, underscoring their sensitivity to chronic cannabis in the fully developed adult female mouse brain. When comparing the plasticity of the brain to chronic cannabis exposure across developmental periods it is interesting to note that we find fewer changes in adolescence, a period of high neuronal plasticity and organization as compared to the fully formed adult brain. Further research is needed to explain this vulnerability with aging and the mechanisms that promote these changes.

## Data availability statement

The original contributions presented in this study are included in the article/Supplementary material, further inquiries can be directed to the corresponding author.

## Ethics statement

The animal study was reviewed and approved by Northeastern University IACUC.

## Author contributions

AT, AN, VV, and MO did the work. PK and CF contributed to the concept, experimental design, drafting, manuscript preparation, and interpretation. All authors contributed to the data generation and analysis.

## Footnotes

1
https://www.nitrc.org/projects/drams

2
https://med.inria.fr/
